# Comparative study of friction between metallic and conventional interactive self-ligating brackets in different alignment conditions

**DOI:** 10.1590/2176-9451.19.3.082-089.oar

**Published:** 2014

**Authors:** Sérgio Ricardo Jakob, Davison Matheus, Maria Cristina Jimenez-Pellegrin, Cecília Pedroso Turssi, Flávia Lucisano Botelho do Amaral

**Affiliations:** 1 MSc in Orthodontics, Camilo Castelo Branco University, UNICASTELO.; 2 MSc in Orthodontics, São Leopoldo Mandic College of Dentistry.; 3 Professor, São Leopoldo Mandic College of Dentistry.; 4 Assistant Professor, Department of Operative Dentistry, São Leopoldo Mandic College of Dentistry.

**Keywords:** Orthodontic brackets, Friction, Esthetics

## Abstract

**Objective:**

The aim of this study was to compare the friction between three bracket models: conventional stainless steel (Ovation, Dentsply GAC), self-ligating ceramic (In-Ovation, Denstply GAC) and self-ligating stainless steel brackets (In-Ovation R, Dentsply GAC).

**Methods:**

Five brackets were used for each model. They were bonded to an aluminum prototype that allowed the simulation of four misalignment situations (n = 10). Three of these situations occurred at the initial phase (in which a 0.016-in nickel-titanium wire was used): 1. horizontal; 2. vertical; and 3. simultaneous horizontal/vertical. One of the situations occurred at the final treatment phase: 4. no misalignment (in which a 0.019 x 0.025-inch stainless steel rectangular wire was used). The wires slipped through the brackets and friction was measured by a Universal Testing Machine.

**Results:**

Analysis of variance followed by Tukey's Test for multiple comparisons (α = 0.05) were applied to assess the results. Significant interaction (p < 0.01) among groups was found. For the tests that simulated initial alignment, Ovation^®^ bracket produced the highest friction. The two self-ligating models resulted in lower and similar values, except for the horizontal situation, in which In-Ovation C^®^ showed lower friction, which was similar to the In-Ovation R^®^ metallic model. For the no misalignment situation, the same results were observed.

**Conclusion:**

The self-ligating system was superior to the conventional one due to producing less friction. With regard to the material used for manufacturing the brackets, the In-Ovation C^®^ ceramic model showed less friction than the metallic ones.

## Abstract (pt)

**Objetivo:**

o objetivo desse trabalho *in vitro* foi comparar o atrito entre três modelos de braquetes: Ovation (convencional, aço inoxidável); In-Ovation C (autoligável, cerâmico) e In-Ovation R (autoligável, aço inoxidável), todos do mesmo fabricante (Dentsply GAC).

**Métodos:**

para cada modelo, foram utilizados cinco braquetes, colados a um protótipo de alumínio, que permitiu a simulação de quatro situações (n = 10), sendo uma delas sem desalinhamento (com utilização de fio retangular de aço inoxidável, com espessuras de 0,019" x 0,025") e outras três com desalinhamento, sendo um horizontal, um vertical e outro simultaneamente combinando ambos (com utilização de fio de níquel-titânio com espessura de 0,016"). O atrito foi mensurado por uma máquina universal de ensaios.

**Resultados:**

os resultados obtidos foram submetidos ao teste de Análise de Variância, complementado pelo teste de comparações múltiplas de Tukey (α = 0,05). Foi observada interação significativa entre os grupos (p < 0,01). Para os ensaios que simularam a fase inicial de alinhamento, realizada com fios de NiTi, o braquete Ovation foi o que produziu o maior atrito, e os dois modelos autoligáveis produziram resultados menores e semelhantes, exceto para o ensaio de desalinhamento horizontal, onde o In-Ovation C apresentou atrito menor do que o similar metálico In-Ovation R. Na fase em que o fechamento de espaço foi simulado, os mesmos resultados foram observados.

**Conclusão:**

pode-se concluir que o sistema de autoligável mostrou-se superior ao convencional, com elastômeros, por produzir menor atrito. Quanto ao material utilizado na confecção dos braquetes, o modelo cerâmico In-Ovation C apresentou menor atrito que os metálicos.

## INTRODUCTION

Over the last few years, one of the most widely studied fields in orthodontic research has been the performance of self-ligating brackets due to its alleged advantages over conventional ligation systems.^1-4^ In addition to retaining the wire inside the bracket slot, the ligation system works in symbiosis with the wire and increases the effectiveness of dental movement.^5^

Friction is represented by the formula: friction = µ x F, in which µ represents the coefficient of friction and F is the perpendicular force when surfaces are in contact. According to Kusy and Whitley,^6^ in order to move the tooth along the arch, the applied force needs to overcome static friction at approximately 50% of the total force applied to the tooth. Thus, high levels of force are needed in situations of high friction. Several articles have shown a significant reduction in friction when self-ligating brackets are used.^7-14^

Another factor directly related to friction at the bracket-wire interface is the material with which the orthodontic accessory is fabricated, since surface roughness directly interferes in the amount of friction produced. Therefore, when choosing the best bracket for dental movement, particularly in mechanics when sliding is a preponderant factor, it is imperative to comparatively assess friction generated by metallic, esthetic, and hybrid brackets (esthetic brackets with metal slots).

In addition, misalignment (horizontal, vertical, horizontal/vertical) or complete alignment may have some type of influence on friction. In horizontal misalignment, friction caused by the ligation system is of primary importance, because in an attempt to displace the wire in the labial direction, there is contact between the wire and the bracket slot and, therefore, direct resistance to the ligation system used. Conversely, in case of vertical misalignment, phenomena such as binding (a region of extremely close contact between the wire and the slot) and notching (the permanent deformation of the wire when it is in contact with the sides of the slots) are more important factors than the ligation system itself when considering the increase of friction.^15,16^ The simultaneous combination of vertical and horizontal misalignments is a situation of crucial importance, since any misalignment may either reduce or add friction. Last, in complete alignment, the use of rectangular wires for sliding movements is considered relevant as they may exert some pressure in the ligation system, a factor that may increase friction.^15^

Therefore, additional research studies are necessary to further investigate not only friction of the ligation systems, but also the composition of orthodontic accessories.

## MATERIAL AND METHODS

### Experimental design

The factors under study in this experiment were as follows:

I) type of bracket: a) metallic self-ligating bracket (In-Ovation R^®^, GAC International Inc., Bohemia, USA); b) ceramic self-ligating bracket (In-Ovation C^®^, GAC International Inc., Bohemia, USA); and c) conventional stainless steel bracket (Ovation®, GAC International Inc., Bohemia, USA).

II) Alignment and leveling situation:

» 1) Horizontal misalignment - 1 mm (0.016-in nickel-titanium wire);
» 2) Vertical misalignment - 1 mm (0.016-in nickel-titanium wire);
» 3) Horizontal/vertical misalignments - 1 mm (0.016-in nickel-titanium wire)
» 4) No misalignment - 0 mm (0.019 x 0.025-in stainless steel wire.

The combination of factors under study resulted in 12 experimental groups (n = 10). The quantitative response variable was the measurement of friction (KgF) obtained in a universal testing machine. Table 1 shows the experimental groups studied.

**Table 1:** Experimental groups.

Bracket	Alignment
In-Ovation R self-ligating metallic bracket (GAC International Inc., Bohemia, USA)	Horizontal malalignment: 1 mm
	Vertical malalignment: 1 mm
	Horizontal/vertical malalignment: 1 mm
	Alignment: 0 mm
In-Ovation C self-ligating ceramic bracket (GAC International Inc., Bohemia, USA)	Horizontal malalignment: 1 mm
	Vertical malalignment: 1 mm
	Horizontal/vertical malalignment: 1 mm
	Alignment: 0 mm
Ovation conventional metallic bracket (GAC International Inc., Bohemia, USA).	Horizontal malalignment: 1 mm
	Vertical malalignment: 1 mm
	Horizontal/vertical malalignment: 1 mm
	Alignment: 0 mm

### Bracket bonding

**Figure 1:**
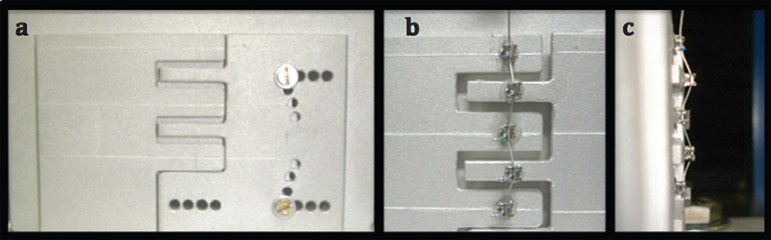
A) Prototype especially designed for the present study. B) Vertical malalignment. C) Horizontal malalignment.

To conduct the tests in this study, a prototype, similar to the one used by Ogata et al^17^ was used. The device enabled bonding of five brackets per test with the purpose of simulating a dental hemi-arch from the central incisor up to the maxillary second pre-molar. It was composed of five winglets designed to bond the brackets. Each one of the winglets had demarcations that allow bonding of the orthodontic accessory in a standardized vertical position. The prototype contained screws for adjustment and spacers that allowed variation of bracket position in horizontal misalignment, as well as in vertical misalignment (Fig 1A).

The prototype face to which the brackets would be bonded was first submitted to airborne aluminum oxide particle abrasion in order to increase bond strength.

Each one of the three bracket groups was composed of five brackets. In the first group, In-Ovation C® esthetic self-ligating brackets were used; in the second group, In-Ovation R^®^ metallic self-ligating brackets; while in the third group, Ovation^®^ conventional metallic brackets were used. All brackets slot measured 0.021 x 0.028-in. Both self-ligating and conventional brackets were used following the Straight-Wire technique and Roth prescription. For the Ovation^®^ model, elastomeric ligatures measuring 1.2 mm in internal diameter were used to tie the wire to the slot (Morelli^®^, Sorocaba, Brazil).

To conduct the tests, the brackets were placed in passive position so as to prevent any possibility of torque, angulation or rotation, which could affect the results. For this purpose, a stainless steel wire 0.021 x 0.025-in guide (Morelli®, Sorocaba, Brazil) was used. The wire guide was set to the testing machine, passing through all the brackets. The orthodontic accessories were then bonded to the winglets of the prototype using Transbond XT^®^ resin (3M Unitek, Monrovia, USA). This resin was light cured by means of Ultralux EL^®^ (Dabi Atlante Indústrias Médico-Odontológicas Ltda, Ribeirão Preto, Brazil) for 40 seconds on each bracket.

### Friction test

The prototype was coupled to the EMIC DL-2000^®^ precision machine (EMIC, São José dos Pinhais, Brazil) and the tests were conducted in dry state. The EMIC machine, with a load cell of 20 KgF, promoted traction of the wire inside the bracket slots affixed to the prototype. Traction was done at a speed of 0.5 mm/min at a trajectory of 1 mm. Data was obtained in KgF.

Four different tests were conducted based on the alignment situations: 1) horizontal misalignment - 1 mm (0.016-in nickel-titanium wire): horizontal variation of brackets at 1 mm, adjusting the position of the prototype by using three 1 mm-thick spacers placed between the mobile piece and base; 2) vertical misalignment - 1 mm (0.016-in nickel-titanium wire): vertical variation of brackets at 1 mm by changing the position of the screws in the corresponding perforations; 3) horizontal/vertical misalignments- 1 mm (0.016-in nickel-titanium wire): a combination of horizontal and vertical misalignments; and 4) No misalignment - 0 mm (0.019 x 0.025-in stainless steel wire): brackets were horizontally and vertically aligned.

Each bracket/wire/adjustment combination of the prototype was tested 10 times, totaling 120 series of tests (n = 10). After each test, new elastomeric ligatures were placed on the conventional brackets by means of Mathieu tweezers. Nickel-titanium and stainless steel wires were replaced after each bracket was tested.

### Statistical analysis

Statistical tests were prepared by the SAS JMP 8^®^ program (SAS Institute Inc., Raleigh, NC, USA), and the Analysis of Variance complemented by Tukey's multiple comparison test with significance level set at 5%.

## RESULTS

Table 2 shows the results obtained for all experimental groups. Significant difference between the bracket group and the simulation of misalignment was found (p < 0.01). In other words, when the position was fixed at 0 mm (using 0.019 x 0.025-in stainless steel wire), the groups with In-Ovation R^®^ and In-Ovation C^®^ self-ligating brackets had the lowest friction values. Although these values were statistically similar among groups, they differed from conventional Ovation brackets (p < 0.01).

**Table 2:** Mean and standard deviation (SD) of friction force expressed in KgF for each experimental group.

Group	Malaligment condition			
	0 mm	1 mm horizontal	1 mm vertical	1 mm horizontal/vertical
	**Mean ± SD**	**Mean ± SD**	**Mean ± SD**	**Mean ± SD**
Ovation	0.340^Ac^ ± 0.024	0.370^Abc ±^ 0.024	0.480^Aa ±^ 0.046	0.410^Ab ±^ 0.041
In-Ovation R	0.154^Bc ±^ 0.061	0.220^Bb ±^ 0.033	0.061^Bd ±^ 0.020	0.370^Aa ±^ 0.024
In-Ovation C	0.158^Ba ±^ 0.031	0.067^Cb ±^ 0.027	0.053^Bb ±^ 0.027	0.132^Ba ±^ 0.018

Means followed by the same letters (upper case in the column and lower case in the line) are not statistically different (p > 0.05).

With regard to horizontal misalignment, all groups presented significant differences. Conventional Ovation^®^ bracket showed the highest friction, followed by In-Ovation R^®^ and In-Ovation C^®^ brackets (p < 0.01).

Lower friction was found in In-Ovation C^®^ self-ligating brackets when the simulation of misalignment in the prototype was modified to 1 mm, vertically. There was no statistically significant difference in comparison to In-Ovation R^®^ bracket. Ovation^®^ bracket showed significantly higher friction (p = 0.01).

In horizontal/vertical misalignment, lower friction was found for In-Ovation C^®^ bracket, which was statistically different in comparison to In-Ovation R^®^ and Ovation^®^ brackets, with higher friction values (p < 0.01).

## DISCUSSION

With a view to providing greater effectiveness in orthodontic mechanics, knowledge about the importance of friction at the bracket-wire interface is one of the most relevant factors and the objective of several research studies.^2,6,13,14,18,19,20^

The aim of this study was to compare friction between self-ligating and conventional ligation systems in different situations: horizontal misalignment which simulates, for example, buccal displacement of teeth; vertical misalignment, which simulates infra-occlusion of maxillary canines^21^ and both clinical situations that may occur together. Additionally, dental alignment (no misalignment), in which only sliding mechanics is needed, was also considered. A specially designed prototype that allowed testing of these different situations was used. The study by Reznikov et al^22^ also used a methodology that allowed horizontal and vertical misalignments. However, their tests did not include simultaneous misalignments, and, therefore, did not reflect the real clinical practice of orthodontic treatment.

When considering self-ligating brackets, the present study opted to use active system composed of a flexible cobalt-chromium clip that undergoes deflections when it is in horizontal contact with the wire. The self-ligating brackets selected were In-Ovation R^®^, metallic model, and its similar esthetic version, In-Ovation C^®^. Both are very similar in design, the only difference is related to the raw material used in their fabrication. In-Ovation R^®^ is made through the system known as MIM (metal-injection-molded), while In-Ovation C^®^ is fabricated with the CIM system (ceramic-injection-molded) with polycrystalline ceramic.^23^ The conventional model (Ovation^®^) used in the tests is also fabricated by the MIM system.

Friction experiments rarely use brackets from the same manufacturer, but oftentimes compare brackets from different manufacturers.^5,8,9,12,14,24,25,26^ On the contrary, the methods employed in this study allowed standardization of the raw materials and technology used for the fabrication of metallic brackets, thus favoring the interpretation of results. Pizzoni et al,^11^ for instance, compared the frictional forces of different brands of self-ligating brackets, and showed that different metal alloys incorporated into the composition of clips may have influenced roughness and, consequently, friction produced by brackets.

In order to simulate a clinical situation, the present study used five brackets forming a hemi-arch from the central incisor up to the maxillary right second pre-molar, similar to the study conducted by Krishnan et al.^10^ In contrast, other studies^27,28^ used only one bracket for each test, which may be a limitation when the objective is to simulate clinical practice as closely as possible. Another important aspect to be discussed is that although the ideal situation was replacing brackets after each test, this was not considered in this study. Each bracket was tested ten times. In spite of that, Kapur et al^29^ observed that there was neither increase nor decrease in friction when brackets were repeatedly used.

The present study used a conventional stainless steel bracket with elastomeric ligatures as control group. Elastomeric ligatures reduce chair time due to being easy to use. Some studies state that elastomeric ligatures produce more friction^30,31^ or similar friction^32^ to stainless steel ones. Thorstenson and Kusy^31^ reported that when angulation exceeds the critical contact angle for binding, the binding component overwhelms the frictional component and the effects of ligature type and method are minimal. Thus, further studies should confirm the effects of ligature type on friction resistance in misalignment situations.

Using a prototype in both misalignment and alignment situations led us to select orthodontic wires with different cross-sections. During the misalignment stages, a 0.016-in nickel-titanium wire was used, similarly to what is recommended by Henao and Kusy.^8^ As for the alignment stage, a 0.019 x 0.025-in wire was used so as to represent the treatment stage in which dental alignment has already been established. Stainless steel alloy is normally recommended for this purpose for presenting less roughness than other alloys.^30,33^

Comparative assessment between the three brackets revealed that the alignment situation showed 50% lower friction in both self-ligating methods, In-Ovation R^®^ and In-Ovation C^®^, in comparison with conventional Ovation® brackets. This result suggests better performance of the self-ligating system, even when esthetic brackets are chosen, and corroborates the findings reported by Voudouris et al.^23^

There was a significant difference in the performance of the three bracket types used for horizontal misalignment at 1 mm, in which In-Ovation C^®^ proved to be superior to In-Ovation R^®^which, in turn, was superior to Ovation^®^. In this case, the self-ligating system showed a distinct advantage over the conventional one. Considering the superiority of In-Ovation C^®^ over In-Ovation R^®^, similar results have been found in the literature.^34,35^ Heo and Baek^35^ observed that Cr-Co clips incorporated to In-Ovation-C^®^ brackets showed more freedom within the bracket slot, thus reducing friction. However, both In-Ovation^®^ models use the same cobalt-chromium clips. Therefore, the difference in friction among them must be attributed to the greater smoothness of the esthetic model.^23^

When the prototype was adjusted to simulate vertical misalignment at 1 mm, In-Ovation C^®^ and In-Ovation R^®^ brackets had statistically similar friction values that were superior to the Ovation^®^ bracket. It is probable that, in addition to the action of the ligation system in friction, binding and notching effects (when the wire is permanently deformed, making sliding difficult) may have decisively influenced sliding mechanics.^36^ Thus, the self-ligating system showed lower friction than the conventional one. It is known that the critical angle (θc), which is the angle (θ) at which the archwire first contacts the edges of the slot, is an important factor that enhances the binding component in in vertical misalignment (or second order angulation).^15^ For self-ligating brackets, the θc is 3.8°, whereas for conventional brackets, the θc is 3.1°.^15^ This condition is especially important for conventional brackets because the θc is easily surpassed, thereby enhancing resistance to sliding. However, when friction resistance is considered, as there was no statistically significant difference between In-Ovation R^®^ and In-Ovation C^®^, binding and notching effects may be more significant than surface roughness of the bracket slot.

Horizontal/vertical misalignment showed that bracket surface roughness is imperative in these conditions, since In-Ovation C^®^ showed less friction than In-Ovation R^®^ and Ovation^®^. Similarity between the two latter metallic brackets showed that, in this situation, the ligation system is not a decisive factor, but rather the raw material used for its fabrication.

After individual assessment of each bracket, Ovation® conventional model showed higher friction when the prototype was misaligned in the vertical direction, only. In the horizontal and horizontal/vertical misalignments, values were respectively lower and similar. The lowest friction values in sliding mechanics were obtained when the brackets were aligned (0 mm) in the prototype. Once again, results suggest that binding and notching effects generated more friction than the ligation system. Apparently, horizontal misalignment reduces binding and notching effects, probably because the wire moves forward from the bottom of the slot, decreasing the vertical effects.

As for In-Ovation R^®^ brackets, higher friction was observed in the horizontal/vertical misalignment, followed by horizontal misalignment, alignment and vertical misalignment. In the case of self-ligating brackets, rigidity of the cobalt-chromium clip showed important effects of friction in situations of horizontal misalignment. It may also be suggested that this rigidity does not allow reduction in binding and notching effects in the horizontal/vertical misalignment, as it occurred with conventional Ovation^®^ brackets. The decisive action of the clip in the aligned position (0 mm) may once again be observed due to the thickness of the wire, 0.019 x 0.025-in. Excessive deflection of the clip occurs because the upper horizontal wall of In-Ovation^®^ is 0.018-in, while the horizontal wall of the wire is 0.025-in. This may cause extreme deflection of the clip, associated with the rigidity of the Co-Cr alloy, which drastically increases friction.

One factor was of paramount importance to explain the results of In-Ovation C^®^ brackets: the clip receives a rhodium coat^34^ that improves its cosmetic value by removing metallic brightness, rendering the clip opaque. According to the manufacturer's instructions, rhodium coat increases the rigidity of cobalt-chromium, which is a characteristic of this alloy when subjected to heat treatment. Due to this increased rigidity, it is hypothesized that the wire causes lower deflection of the clip. Thus, because the clip is not deflected in the horizontal misalignment; the 0.016-in wire has more space inside the slot, which results in significantly reduced friction in comparison to the same situation experienced by In-Ovation R^®^. Voudouris et al^23^ demonstrated that the lower friction promoted by In-Ovation C^®^ may be attributed to the new ceramic-injected molding technique that produced a smoother, glass-like slot in In-Ovation- C^®^. Heo and Baek^35^ observed that the In-Ovation C^®^ clip has a rounded shape that may result in lower seating forces and lower friction. Nevertheless, all these hypotheses need to be further clarified.

It is important to point out that friction in vertical misalignment did not cause a great alteration in In-Ovation C^®^ due to the rigidity of the clip. Another interesting result may also be observed in the alignment of the prototype brackets. There was a significant increase in friction when the 0.019 x 0.025-in wire was used, probably because there was no gap in the wire inside the slot, as occurred when the 0.016-in wire was used. In this situation, friction in In-Ovation C^®^ bracket was similar to that found for In-Ovation R^®^.

Thus, both ligation system and composition might decisively influence tooth movement in situations of horizontal, vertical and vertical/horizontal misalignments, as well as during sliding mechanics, since they directly influence friction.

## CONCLUSION

It is suggested that:

» In-Ovation R^®^ and In-Ovation C^®^ self-ligating bracket systems showed lower friction at all alignment levels tested in this study in comparison with the conventional Ovation^®^ bracket system, except for the horizontal/vertical misalignment in which In-Ovation R^®^ showed similar friction in comparison to the Ovation^®^ bracket.
» Friction was similar for self-ligating metallic and ceramic brackets, except for horizontal and horizontal/vertical misalignments, in which the ceramic model showed lower friction.
